# The Ratio of Oxidized Lipoprotein(a) to Native Lipoprotein(a) and the Endothelial Function in Patients with Type 2 Diabetes Mellitus

**DOI:** 10.3390/ijms20194909

**Published:** 2019-10-03

**Authors:** Kazuhiko Kotani, Shingo Yamada, Hirokazu Takahashi, Yoshitaka Iwazu, Toshiyuki Yamada

**Affiliations:** 1 Division of Community and Family Medicine, Department of Clinical Laboratory Medicine, Jichi Medical University, Shimotsuke-City, Tochigi 329-0498, Japan; 2 Department of Clinical Laboratory Medicine, Jichi Medical University, Shimotsuke-City, Tochigi 329-0498, Japan; iwazu@jichi.ac.jp (Y.I.); yamadanji@jichi.ac.jp (T.Y.); 3 Central Laboratory, Shino-Test, Sagamihara-City, Kanagawa 252-0331, Japan; shingo.yamada@shino-test.co.jp; 4 Division of Metabolism and Endocrinology, Faculty of Medicine, Saga University, Saga-City, Saga 849-8501, Japan; takahas2@cc.saga-u.ac.jp

**Keywords:** endothelium, atherosclerosis, Endo-PAT, oxidative stress, reactive hyperemia, Lp(a)

## Abstract

The ratio of oxidized lipoprotein(a) to native lipoprotein(a) (oxLp(a)/Lp(a)) may be a reasonable index for assessing endothelial dysfunction in type 2 diabetes mellitus (T2DM). The present study investigated whether the oxLp(a)/Lp(a) level is correlated with the endothelial function using the Endo-PAT^TM^, a newly developed device, in patients with T2DM. A total of 63 patients with T2DM (mean age: 59 years old) were enrolled in the study. The patients’ serum Lp(a) and oxLp(a) levels were measured using an enzyme-linked immunosorbent assay. The reactive hyperemia index (RHI) level was measured using an Endo-PAT^TM^ 2000. A correlation analysis between the measured variables was conducted. Among the patients, the mean hemoglobin A1c was 7.8%. The median level of oxLp(a)/Lp(a) was 0.28 (interquartile range: 0.07–0.54), and the mean RHI was 1.8 (standard deviation: 0.4). In a multiple linear regression analysis, the oxLp(a)/Lp(a) level was an independent, significant, and inverse variable for the RHI level (*β* = −0.26, *p* < 0.05), along with male gender. A high oxLp(a)/Lp(a) level may reflect endothelial dysfunction, as assessed by the Endo-PAT^TM^, in patients with T2DM. Further studies are warranted to confirm the observed findings.

## 1. Introduction

Patients with type 2 diabetes mellitus (T2DM) have a high risk of cardiovascular disease (CVD) caused by atherosclerosis [1]. Endothelium regulates the vascular tone and growth in communication with circulating blood molecules relating to inflammation, oxidation, immunity, and thrombosis [2,3,4]. Endothelial dysfunction increases the risk of future CVD events and endothelial dysfunction is an indicator of earlier atherosclerosis [2,3,4]. The assessment of endothelial dysfunction is crucial in the clinical setting for preventing and managing the development of CVD in T2DM.

Lipoproteins are factors involved in endothelial dysfunction [5,6]. Low-density lipoprotein (LDL), especially upon modification, is a well-known factor detected in the subendothelial space with subsequent foam cell formation [5,6]. One particular lipoprotein species, lipoprotein(a) (Lp(a), an LDL-like particle) increases the risk of CVD when the circulating blood level is high in the general population [7,8], and some studies have indicated a similar risk of Lp(a) for CVD in patients with T2DM [9,10]. While its physiological role is not completely understood, Lp(a) can contribute to endothelial dysfunction by activating monocytes and exerting atherothrombotic properties [6,7]. In addition to the native Lp(a), its form modified by oxidation, oxidized Lp(a) (oxLp(a)) is also hypothesized to be positively associated with atherosclerotic processes as oxidative stress induces and enhances the processes [7]. Indeed, the clinical significance of the measurement of oxLp(a) on several atherosclerotic pathologies has been reported [11,12,13].

T2DM is an oxidative stress condition [14] in which oxLp(a) is likely to manifest [11]. The liver produces Lp(a) which is then released to the circulation. Of interest, insulin modulates the synthesis of Lp(a) in the liver and reduces the Lp(a) level [15,16]. T2DM can induce a high insulin level/insulin resistance and a reduced Lp(a) level [15,16]. The ratio of oxLp(a) to native Lp(a) (oxLp(a)/Lp(a)) may therefore be a reasonable index reflecting endothelial dysfunction in T2DM patients. However, the utility of this index in such patients has not yet been investigated.

Methods of easily evaluating endothelial function have been explored [2,3], but the application of conventional ultrasonic devices has practical limitations, such as depending on an individual operator’s technique. The Endo-PAT^TM^ (peripheral arterial tone, Itamar Medical) is a recently developed device that involves an operator-independent technique, allowing for the automatic and noninvasive measurement of endothelial function via reactive hyperemia [2,3]. The present study investigated whether or not the oxLp(a)/Lp(a) level was correlated with the endothelial function using the Endo-PAT in patients with T2DM.

## 2. Results

The clinical characteristics of patients are listed in Table 1. The study patients were 41 men and 22 women (mean age: 59 years old). Their mean hemoglobin A1c level was 7.8% (standard deviation: 2.1). The median level of Lp(a) was 0.46 mmol/L (IQR: 0.24–0.86), that of oxLp(a) was 0.11 nmol/L (IQR: 0.04–0.28), and that of oxLp(a)/Lp(a) was 0.28 (IQR: 0.07–0.54). The mean RHI was 1.8 (standard deviation: 0.4).

**Figure 1:**
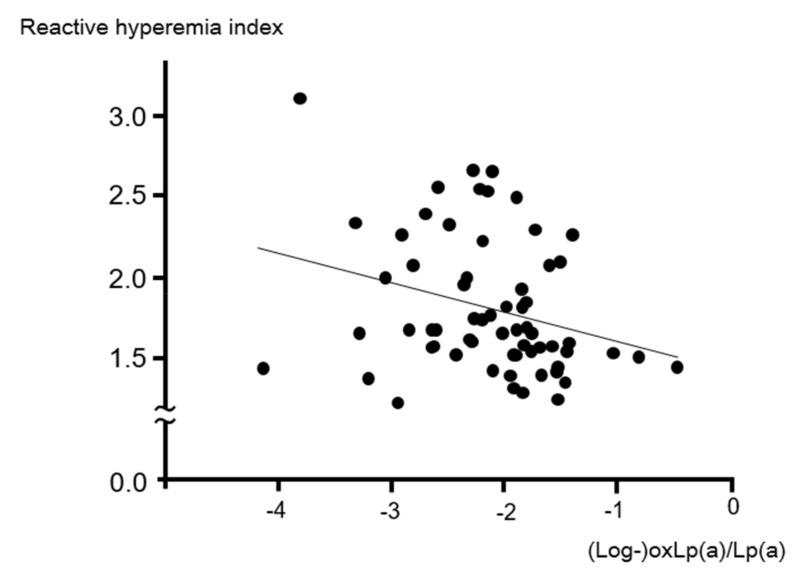
Correlation between the oxLp(a)/Lp(a) and reactive hyperemia index.

As shown in Table 2, Pearson’s correlation analysis showed the oxLp(a)/Lp(a) level to be significantly and inversely correlated with the RHI (*r* = −0.29, *p* = 0.02, Figure 1). The RHI was correlated insignificantly but positively with the Lp(a) level, and insignificantly but inversely with the oxLp(a) level. Adjusted for confounders, a stepwise multiple linear regression analysis revealed that the oxLp(a)/Lp(a) level was an independent, significant, and inverse variable for the RHI (*β* = −0.26, *p* = 0.04), along with male gender.

We performed a sub-analysis of the correlation between the oxLp(a)/Lp(a) and RHI using groups divided by comparatively high and low values of cardiometabolic variables based on the mean/median value of the variables. The results of the oxLp(a)/Lp(a)-RHI correlations were as follows: the group with a high level of body mass index (*r* = −0.25, *p* > 0.05) and its low level (*r* = −0.37, *p* = 0.04, but it did not remain to be significant in a stepwise multiple linear regression analysis), the group with a high level of blood pressure (*r* = −0.32, *p* > 0.05) and its low level (*r* = −0.36, *p* > 0.05), the group with a high level of total cholesterol (*r* = −0.35, *p* > 0.05) and its low level (*r* = −0.21, *p* > 0.05), the group with a high level of high-density lipoprotein cholesterol (*r* = −0.20, *p* > 0.05) and its low level (*r* = −0.34, *p* > 0.05), and the group with a high level of hemoglobin A1c (*r* = −0.33, *p* > 0.05) and its low level (*r* = −0.23, *p* > 0.05). Among the sub-analyses, a marked difference was noted between the groups with a high and low level of triglyceride. The oxLp(a)/Lp(a) level was significantly and inversely correlated with the RHI (*r* = −0.40, *p* = 0.03; *β* = −0.38, *p* = 0.02) in the group with a high triglyceride level, while the oxLp(a)/Lp(a) level was insignificantly correlated with the RHI (*r* = −0.17, *p* > 0.05) in the group with a low triglyceride level.

## 3. Discussion

As assessed by the Endo-PAT^TM^, the present study revealed that a high oxLp(a)/Lp(a) level could indicate an impaired endothelial function in patients with T2DM. Thus, the oxLp(a)/Lp(a) may be a more sensitive index than the Lp(a) and oxLp(a) alone for detecting endothelial dysfunction in this population. The use of the oxLp(a)/Lp(a) for detecting the development of endothelial dysfunction in T2DM would be meaningful, as the endothelial function is not easily assessed in the daily clinical setting.

As described in the Introduction section, increased oxLp(a) and reduced Lp(a) levels are expected to result in a low RHI level (indicative of endothelial dysfunction) as T2DM is an oxidative stress condition and insulin resistance-related pathology that reduces Lp(a) synthesis [14,15,16]. An inverse oxLp(a)-RHI correlation or a positive Lp(a)-RHI correlation was observed, but it was weak (insignificant) in the present study. The relatively homogenous or non-diverse characteristics of the present study population (i.e. all were patients suffering from T2DM, and the variation of hemoglobin A1c or RHI was not very large among patients) might have led to a not-fully-significant correlation between the two variables. In such a situation, considering the oxidative level per Lp(a) as the oxLp(a)/Lp(a) could be effective to see the correlation with the RHI.

In the sub-analysis, there was a greater correlation between the oxLp(a)/Lp(a) and RHI in the group with a high triglyceride level relative to the group with a low triglyceride level. The triglyceride level is thought to be a relevant surrogate marker for insulin resistance in T2DM [17]. This could partly explain why the correlation between the oxLp(a)/Lp(a) and RHI was enhanced in the high-triglyceride group. Though the result was obtained by a small sub-analysis, it may indicate a condition to consider the use of the oxLp(a)/Lp(a).

The observation that male gender was correlated with a reduced RHI in the present study is of note. Some reports have described an increased risk of CVD in women compared with men among T2DM patients, although the reasons remain unclear [18,19]. However, no gender-based differences in the development of CVD events among T2DM patients have been reported in Japanese studies [19,20]. Such differences between countries in the association of the DM state with the CVD risk merit further research.

This study had several limitations. The sample size was relatively small. This was a cross-sectional study, and the causality could not be inferred. The simultaneous measurement of other additional markers, especially those relating to oxidative stress and the endothelial function, might expand our understanding of this phenomenon. Addressing these issues will be our next challenge.

In summary, a high oxLp(a)/Lp(a) level may reflect endothelial dysfunction, as assessed by the Endo-PAT^TM^, in patients with T2DM. Further studies are warranted to confirm the clinical application of oxLp(a)/Lp(a) for managing the CVD risk in this population.

## 4. Methods

This study included 63 patients with T2DM who were free of ischemic heart disease. T2DM was defined as having a blood hemoglobin A1c of ≥ 6.5%, fasting glucose of ≥ 7.0 mmol/L, and/or taking glucose-modulating medication (definition by Japan Diabetes Society [21] and American Diabetes Association [22]). The institutional ethics committee approved the study (No. 12-031; 2012) and each patient gave their informed consent.

All data were obtained from patients in an overnight fasted state. The body mass index was determined based on the weight and height measured while the patients wore light clothing. The seated systolic/diastolic blood pressure (by which the mean blood pressure was determined) was measured in the upper arm. Blood levels of glucose and lipids were measured enzymatically. The hemoglobin A1c level was measured by a high-performance liquid chromatographic method. The serum Lp(a) and oxLp(a) levels were measured using an enzyme-linked immunosorbent assay, as described previously [11,12,13]. For the assay of oxLp(a), the oxLp(a)-specific monoclonal antibody (161E2), which detects an epitope that presents in kringle-IV type-2 of apo(a) with oxidative modification and thus reacts with only oxLp(a), was used as the solid-phase antibody and the detecting capture antibody. In the actual procedure, microtiter plates were coated with the anti-oxLp(a) antibody in phosphate-buffered saline (PBS) and incubated at 37 °C. After incubating the plates with PBS containing 1% bovine serum albumin, the serum samples were added to the wells. The plates were incubated at room temperature with the anti-oxLp(a) antibody conjugated to peroxidase, and TM-Blue was added to the wells. The stop solution was used to stop the enzyme reaction, and the absorbance was then read at 450 nm by a micro-plate reader machine. The intra and inter-assay coefficients of variation were 1.2% and 5.0%.

The reactive hyperemia index (RHI) level was measured in the dorsal position using arteries of both arms with fingertips by an Endo-PAT^TM^ 2000 (Itamar Medical, Caesarea, Israel) [2,3]. Hyperemia was induced using a cuff that occluded blood flow through the brachial artery on one hand for five minutes. The response to the reactive hyperemia was calculated using the response level pre and post-occlusion. The index was normalized using the level of the contra-lateral arm as the control. A low RHI level indicates an impaired endothelial function.

Data are shown as the mean ± standard deviation or the median plus the interquartile range (IQR). Pearson’s correlation analysis and a stepwise multiple linear regression analysis were used to evaluate the correlations between the variables and the RHI. Skewed variables (triglyceride, Lp(a), oxLp(a), oxLp(a)/Lp(a)) were log-transformed for the analyses. A *p* value < 0.05 was significant.

## Figures and Tables

**Table 1:** Clinical characteristics.

Variable	Mean ± SD/Median (lQR)
Age (years)	59 ± 12
Gender (men/women)	41/22
Smoker (*n*)	17 (27%)
Body mass index (kg/m^2^)	26.3 ± 2.7
Mean blood pressure (mmHg)	93.7 ± 11.5
Total cholesterol (mmol/L)	5.35 ± 1.09
HDL cholesterol (mmol/L)	1.39 ± 0.43
Triglyceride (mmol/L)	1.45 (1.05–2.15)
Glucose (mmol/L)	7.89 ± 2.73
Hemoglobin A1c (%)	7.8 ± 2.1
Lp(a) (mmol/L)	0.46 (0.24–0.86)
OxLp(a) (nmol/L)	0.11 (0.04–0.28)
OxLp(a)/Lp(a) (ratio)	0.28 (0.07–0.54)
Reactive hyperemia index	1.8 ± 0.4

SD: standard deviation, IQR: interquartile range, HDL: high-density lipoprotein, Lp(a): lipoprotein (a), OxLp(a): oxidized Lp(a).

**Table 2:** The correlation between the variables and reactive hyperemia index.

Variable	*r (p)*	*β (p)*
Age (years)	0.01 (0.99)	NE
Gender (men)	−0.29 (0.02)	−0.25 (0.04)
Smoker	0.03 (0.81)	NE
Body mass index (kg/m^2^)	−0.04 (0.73)	NE
Mean blood pressure (mmHg)	−0.10 (0.46)	NE
Total cholesterol (mmol/L)	0.05 (0.69)	NE
HDL cholesterol (mmol/L)	0.09 (0.50)	NE
Triglyceride (mmol/L)	0.03 (0.83)	NE
Glucose (mmol/L)	−0.21 (0.10)	NE
Hemoglobin A1c (%)	−0.16 (0.20)	NE
Lp(a) (mmol/L)	0.13 (0.30)	NE
OxLp(a) (nmol/L)	−0.19 (0.14)	NE
OxLp(a)/Lp(a) (ratio)	−0.29 (0.02)	−0.26 (0.04)

HDL: high-density lipoprotein, Lp(a): lipoprotein (a), OxLp(a): oxidized Lp(a), NE: not extracted in stepwise regression model. *r*: Pearson’s correlation coefficient, *β*: stepwise multiple regression coefficient. Nonparametric variables were calculated after a log-transformation. Significance level: *p* < 0.05.
